# Biofilm Morphotypes and Population Structure among *Staphylococcus epidermidis* from Commensal and Clinical Samples

**DOI:** 10.1371/journal.pone.0151240

**Published:** 2016-03-15

**Authors:** Llinos G. Harris, Susan Murray, Ben Pascoe, James Bray, Guillaume Meric, Leonardos Magerios, Thomas S. Wilkinson, Rose Jeeves, Holger Rohde, Stefan Schwarz, Herminia de Lencastre, Maria Miragaia, Joana Rolo, Rory Bowden, Keith A. Jolley, Martin C. J. Maiden, Dietrich Mack, Samuel K. Sheppard

**Affiliations:** 1 Medical Microbiology & Infectious Diseases, Institute of Life Science, Swansea University Medical School, Swansea University, Swansea, United Kingdom; 2 MRC CLIMB Consortium, Institute of Life Science, Swansea University, Swansea, United Kingdom; 3 Department of Zoology, University of Oxford, Oxford, United Kingdom; 4 Institut für Medizinische Mikrobiologie, Virologie & Hygiene, Universitätsklinikum Hamburg-Eppendorf, Universität Hamburg, Hamburg, Germany; 5 Institute of Farm Animal Genetics, Friedrich-Loeffler-Institut, Neustadt-Mariensee, Germany; 6 Laboratory for Molecular Genetics, Instituto de Tecnologia Química e Biológica, Oeiras, Portugal; 7 Laboratory of Microbiology, The Rockefeller University, New York, New York, United States of America; 8 Laboratory of Bacterial Evolution and Molecular Epidemiology, Instituto de Tecnologia Química e Biológica António Xavier, Oeiras, Portugal; 9 The Wellcome Trust Centre for Human Genetics, Oxford Genomics Centre, Oxford, United Kingdom; 10 Institut für Medizinische Diagnostik GmbH, Mikrobiologie/Infektiologie, Ingelheim, Germany; Institut Pasteur, FRANCE

## Abstract

Bacterial species comprise related genotypes that can display divergent phenotypes with important clinical implications. *Staphylococcus epidermidis* is a common cause of nosocomial infections and, critical to its pathogenesis, is its ability to adhere and form biofilms on surfaces, thereby moderating the effect of the host’s immune response and antibiotics. Commensal *S*. *epidermidis* populations are thought to differ from those associated with disease in factors involved in adhesion and biofilm accumulation. We quantified the differences in biofilm formation in 98 *S*. *epidermidis* isolates from various sources, and investigated population structure based on ribosomal multilocus typing (rMLST) and the presence/absence of genes involved in adhesion and biofilm formation. All isolates were able to adhere and form biofilms in *in vitro* growth assays and confocal microscopy allowed classification into 5 biofilm morphotypes based on their thickness, biovolume and roughness. Phylogenetic reconstruction grouped isolates into three separate clades, with the isolates in the main disease associated clade displaying diversity in morphotype. Of the biofilm morphology characteristics, only biofilm thickness had a significant association with clade distribution. The distribution of some known adhesion-associated genes (*aap* and *sesE*) among isolates showed a significant association with the species clonal frame. These data challenge the assumption that biofilm-associated genes, such as those on the *ica operon*, are genetic markers for less invasive *S*. *epidermidis* isolates, and suggest that phenotypic characteristics, such as adhesion and biofilm formation, are not fixed by clonal descent but are influenced by the presence of various genes that are mobile among lineages.

## Introduction

Hospital acquired infections associated with implanted biomaterials such as intravenous catheters, joint prostheses, shunts and heart valves cause substantial morbidity and mortality [1]. Coagulase-negative staphylococci (CoNS), mainly *Staphylococcus epidermidis*, are currently the most frequent cause of such medical device-associated infections [2], responsible for an estimated 1.7 million infections in the US each year and 100,000 deaths [3, 4], at an estimated annual cost of $35–45 billion [5]. A major concern of biomedical device-related infections is their chronic persistence due to biofilm formations, and failure to respond to antibiotics, often necessitating the removal of the device associated with the infection.

A major challenge to reduce the burden of nosocomial infection is to identify if certain *S*. *epidermidis* lineages have a greater pathogenic potential than others. DNA sequencing techniques, including multi-locus sequence typing (MLST) and whole genome sequencing have been instructive in showing that commensal and clinical *S*. *epidermidis* comprise of highly diverse assemblages of related strains [2, 6]. However, the factors involved in generating and maintaining this genetic structure, and how they relate to pathogenicity-associated phenotypes, are poorly understood. For example, closely related isolates can display different phenotypes, and among the most characterized differences between isolates from the skin and infected sites is a greater propensity for biofilm formation among clinical isolates [7]. Such differences may allow them to make the transition from the commensal skin environment to implant-associated infections. The formation of complex adherent bacterial biofilms involves four steps: (i) primary attachment; (ii) accumulation; (iii) maturation; and (iv) detachment. The initial adhesion to an implant surface involves cell-wall-anchored (CWA) proteins/adhesins, such as the microbial surface components recognising adhesive matrix molecules (MSCRAMMs) [8], that are responsible for binding the bacteria directly to inert surfaces or to the host’s extracellular proteins coating the surface. *S*. *epidermidis* adhesins to fibrinogen (Fbe/SdrG), fibronectin (Embp), collagen (SdrF), vitronectin (AtlE, Aae) and elastin (EbpS) have all been identified in *S*. *epidermidis* [9–12]. Three independent mechanisms of biofilm accumulation have been identified. The first is mediated by the polysaccharide intercellular adhesin (PIA), synthesised by *icaADBC* encoded proteins [13, 14]. The other two, mediated by proteinaceous factors independent of the *icaADBC* locus, involve the accumulation associated protein (Aap) [15], and the extracellular matrix-binding protein (Embp) [16]. However, little is known about the extent to which variations in biofilm phenotypes are the result of widespread acquisition of known adhesins and other proteinaceous factors, or clonal expansion of specific lineages.

Here, we characterize variation in 117 *S*. *epidermidis* isolates by genotyping using Multi Locus Sequence Typing (rMLST) [17], an approach which indexes variation of the 53 genes encoding the bacterial ribosome protein subunits (*rps and rpl* genes). This has been compared to phenotypic variation in biofilm formation as quantified by confocal microscopy. Using these data, our aim was to investigate (i) the level of variation in biofilm phenotype in *S*. *epidermidis* isolates; (ii) the correlation between genotypic groupings (clade, clonal complex and sequence type), known biofilm associated genes and biofilm phenotype classification, and (iii) the extent to which the phenotype varies among closely related *S*. *epidermidis* isolates and to what extent this is linked to isolate source (commensal and clinical).

## Materials and Methods

### Bacterial isolates

*S*. *epidermidis* isolates (n = 117) were collected from clinical and commensal sites (Table 1). Clinical isolates were obtained from ENDO-Klinik Hamburg, and University Hospital Hamburg-Eppendorf, Germany [18, 19]; and the Laboratory of Molecular Genetics of the Instituto de Tecnologia Química e Biológica António Xavier, Universidade Nova de Lisboa, Portugal [6, 20]. The commensal isolates consisted of human nasal mucosa and skin surface isolates collected from healthy volunteers at Swansea University, and animal isolates from the Institute of Farm Animal Genetics, Friedrich-Loeffler-Institut, Germany [21]. All isolates were streaked from frozen stocks onto Columbia agar supplemented with 5% horse blood (E&O Laboratories Ltd, Bonnybridge, UK), and incubated overnight at 37°C; and then used in the subsequent experiments.

**Table 1:** List of the *S*. *epidermidis* isolates used in the study.

Isolate source		No. isolates	Reference
Clinical	Prosthetic joint infections	38	18
	Catheter infections	26	19
	Other infections	6	6
Commensal	Nasal mucosa	20	This study
	Skin surface	20	This study
	Animal	7	21

### Biofilm morphotype analysis

Characteristics of the biofilm phenotype of the *S*. *epidermidis* isolates were determined using confocal laser scanning microscopy (CLSM) and image analysis, as in previous studies [22–25]. Briefly, each isolate was cultured from a 3 h pre-culture into an 8-well chamber slide (ibidi GmbH, Planegg-Martinsried, Germany) to a starting OD_600_ of 0.05, and incubated stationary at 37°C for 20–24 h. Non-adherent bacteria were then removed by washing the wells with phosphate buffered saline and the adherent bacteria stained with SYTO9 (Life Technologies Ltd, Paisley, UK), before being visualised with a Zeiss LSM 510-META Confocal laser scanning microscope and Zen software (Carl Zeiss, Jena, Germany). Four image stacks were taken randomly from three independent samples per isolate (12 stacks in total). The image stacks were then analysed using the COMSTAT software [23]. A fixed threshold value and connected volume filtration was used for all image stacks; and the biofilm parameters average biofilm thickness (a measure of the spatial size of the biofilm in μm), biovolume (the volume (μm^3^) of the biomass per μm^2^ of substratum area; μm^3^ per μm^2^) and roughness coefficient (Ra, correlates with biofilm heterogeneity and measured in μm) were determined for each image stack [23]. Regression analysis was used to estimate the relationship between biovolume, thickness and Ra, and Fisher’s exact test was used to examine the significance in the association between biovolume, thickness and Ra measurements, with the level of significance set at *p* ≤ 0.05. An agglomerative hierarchical cluster analysis (AHC) was used to examine the relationship between biovolume, thickness, Ra for the biofilms formed by the *S*. *epidermidis* isolates. The AHC was an unweighted pair-group average agglomeration with Euclidean distance for dissimilarity. From the COMSTAT measurements, the AHC dendrogram and CLSM images, it was possible to group the isolates into 5 biofilm structure patterns as defined in Table 2.

**Table 2:** Definitions of the biofilm structure patterns described in this study.

Biofilm structure pattern	Thickness (μm)	Biovolume (μm^3^ per μm^2^)	Ra (μm)
dense thick uneven	> 10	> 10	0.04–0.20
dense thick smooth	> 10	> 10	< 0.1
patchy thin smooth	< 10	< 10	< 0.1
patchy thin rough	< 10	< 10	< 0.2
patchy thin uneven	< 10	< 10	0.04–0.20

### DNA extraction and sequencing

For DNA extraction, the 117 *S*. *epidermidis* isolates were grown in 2 ml peptone yeast (PY) broth overnight at 37°C, harvested by centrifugation, and chromosomal DNA extracted using a Qiagen QiAmp DNA mini kit (Qiagen, Crawley, UK) following the manufacturer’s instructions using 1 μg/mL lysostaphin (Sigma-Aldrich, Gillingham, UK) to facilitate cell lysis. DNA was quantified using a Nanodrop spectrophotometer, and the Quant-iT DNA Assay Kit (Life Technologies, Paisley, UK) before sequencing. High-throughput genome sequencing was performed using a HiSeq 2500 machine (Illumina, San Diego, CA, USA) and the 100bp short read paired-end data was assembled using the *de novo* assembly algorithm within *Velvet* software [26] (version 1.2.08). Resulting data was then archived in the Staphylococcal Bacterial Isolate Genome Sequence database (BIGSdb) [27]. This software uses a gene-by-gene approach for genome alignment and comparison supported by the BLAST algorithm, for identification of allelic orthologs, and file generation for investigating phylogenetic networks, and locus information for the investigation of gene function. Isolate information, including genome sequence files are publically available (Dryad, doi: 10.5061/dryad.82jq4 for the 14 previously published isolates and doi: 10.5061/dryad.br0h8 for the 103 other isolates).

### Genotype analysis

The *S*. *epidermidis* RP62A reference genome [28] was used as a basis for defining locus designations, and reference sequences for each of these were stored in BIGSdb (http://zoo-talisker.zoo.ox.ac.uk/perl/bigsdb/bigsdb.pl?db=staphylococcus). Isolate genomes, stored as concatenated contiguous sequence files—including gaps for missing nucleotides (or entire genes), were aligned on a gene-by-gene basis using MUSCLE software [29], and the BLAST algorithm was used to identify gene orthologs at all loci in the reference genome. These were defined as reciprocal best hits for a sequence with ≥70% nucleotide identity and a 50% difference in alignment length.

The rMLST approach was used to investigate the genetic relationship between the 117 isolates [17]. Orthologs for the 53 genes encoding the bacterial ribosome protein subunits (*rps and rpl* genes) were defined in all isolates by comparison to the annotated genome of *S*. *epidermidis* RP62A [17]. Reciprocal best hits identifying 11,168 *rps* and *rpl* alleles were identified using BLAST as described above. CLONALFRAME, a model-based approach to determining microevolution in bacteria, was used to estimate the genealogies for these alignments [30]. This program differentiates mutation and recombination events on each branch of the tree based on the density of polymorphisms. Clusters of polymorphisms were likely to have arisen from recombination, and scattered polymorphisms were considered likely to have arisen from mutation. The program was run with 50,000 burn-in iterations, followed by 50,000 sampling iterations. The consensus tree represents combined data from three independent runs with 75% consensus required for inference of relatedness. Recombination events were defined as sequences with a length of >50 bp with a probability of recombination ≥75% over the length, reaching 95% in at least one site.

### Presence of biofilm associated genes

The presence of known biofilm-associated genes was investigated by BLAST comparison to a reference genome [27, 31, 32]. First, a reference gene list was assembled from the publicly available *S*. *epidermidis* strain RP62A genome [28] (GenBank: NC_002976), including genes that have been shown to be associated with *S*. *epidermidis* adhesion and biofilm formation. Adhesion and biofilm-associated genes including *aae*, *aap*, *atlE*, *bhp*, *embp*, *ebpS*, *fbe/sdrG*, *icaADBC*, *sdrF*, *sesC*, *sesE*, *sesG*, *sesH* and *sesI* [9–16, 33, 34], were considered as being present when a BLAST match with a >70% nucleotide sequence identity on ≥50% of sequence length was recorded. Where present, genes where mapped onto the CLONALFRAME tree to examine the significance of association between the clonal frame substructure and the presence of a specific gene. Data were analysed statistically by Fisher’s exact test and Chi-square, with the level of significance set at *p* ≤ 0.05.

## Results

### Clonal structure based on rMLST genes

**Fig 1:**
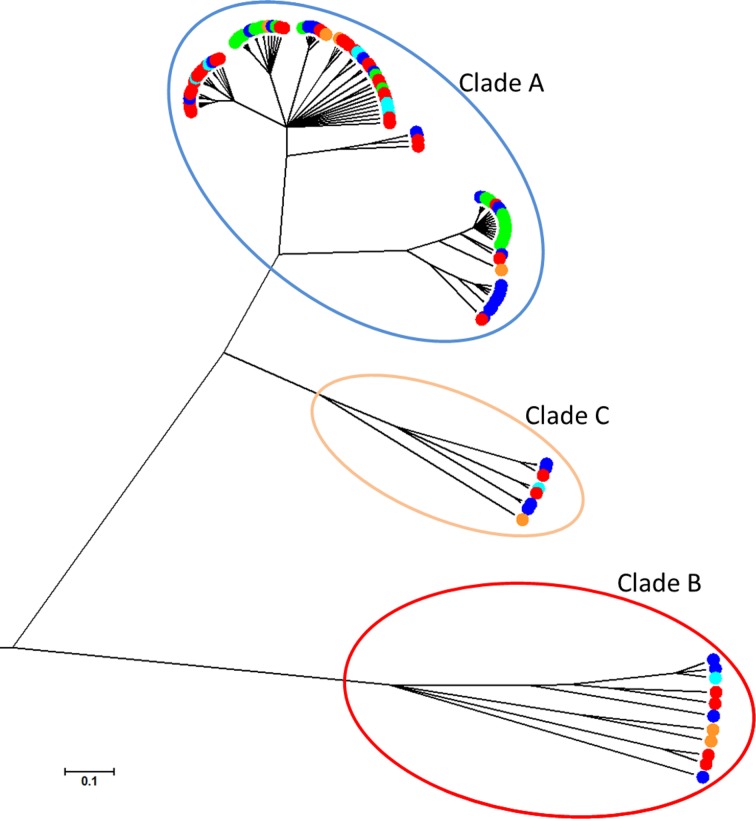
Population structure of 117 S. epidermidis isolates constructed from 53 rMLST genes and implemented in CLONALFRAME, with the 3 clades highlighted. The scale (0.1) represents the number of substitutions per site. Isolates are coloured according to source: PJI (blue); catheter (green); undefined infection (orange); healthy nasal and skin (red); animal (turquoise).

The rMLST locus genealogy, estimated using CLONALFRAME, showed that the *S*. *epidermidis* isolates clustered into 3 clades (Fig 1), as previously described [32]. Commensal and pathogenic isolates were distributed in multiple sequence clusters (*p* ≤ 0.05). Clade A comprised of 98 isolates that clustered into three groups, and contained isolates belonging to 7-locus MLST clonal complexes 2, 5, 89 and 291 [6]; whilst the isolates in Clades B (11 isolates) and C (8 isolates) did not belong to any previously described clonal complexes. There was a significant association between clonal complex and sub-clade sequence-cluster distribution (*p* ≤ 0.001, S1 Table). For example CC291 corresponded to a single lineage; however, some clonal complexes were represented by isolates in separate sequence clusters including CC2, CC5 and CC8. There was a significant association between clonal complex and isolate source (*p* = 0.001), with 54% of infection isolates belonging to CC2 compared to only 9% from healthy carriage and none in the animal isolates. CC5 and CC89 were more represented among isolates from healthy carriage constituting 31% and 28% of the isolates from this source respectively.

### Biofilm morphotype analysis

**Fig 2:**
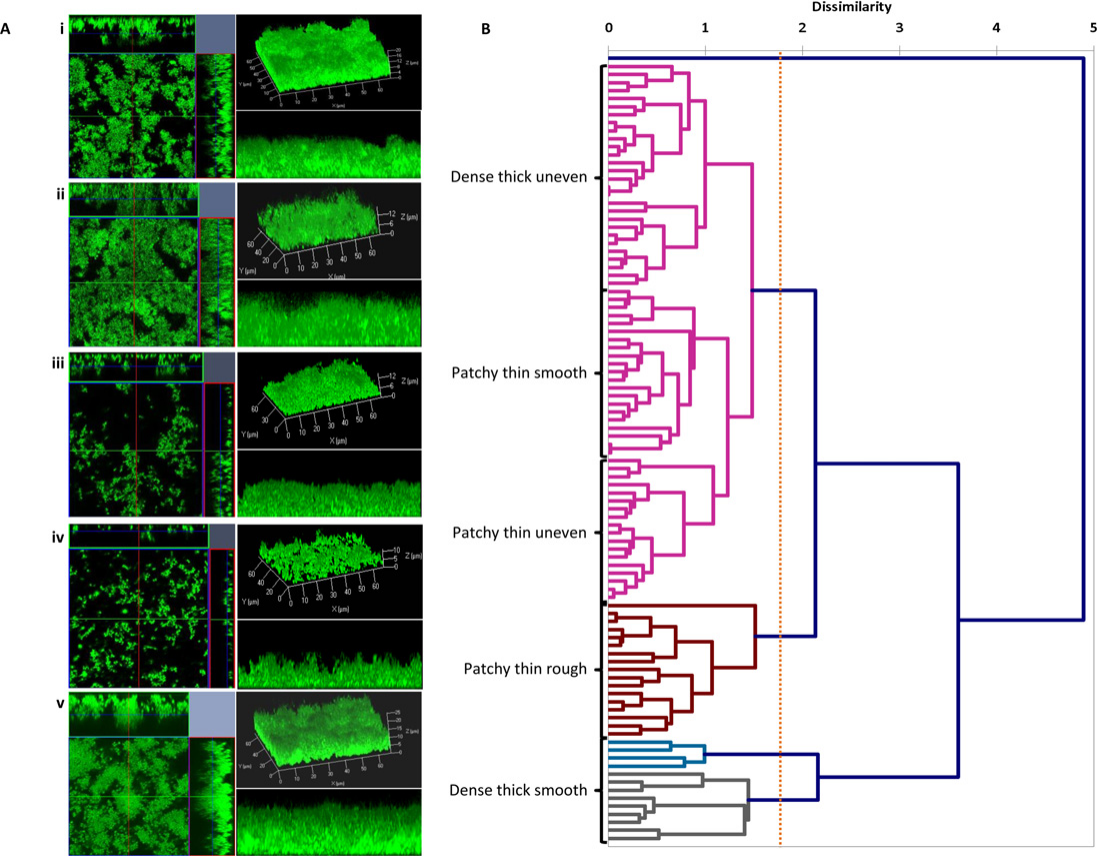
Confocal laser scanning microscopy (CLSM) biofilm images from different isolates and agglomerative hierarchical clustering (AHC) dendrogram labelled with biofilm morphotypes. (A) Example of CLSM images comprising orthogonal view of Z-stacks (left panel), 3D image of the Z-stacks (top right panel), and 3D cross-section of Z-stacks (bottom right panel). Scale of in μm. (B) Each set of images corresponds to a biofilm structure group defined according to CLSM measurements (average thickness and roughness coefficient) (i) dense thick uneven; (ii) patchy thin smooth; (iii) patchy thin uneven; (iv) patchy thin rough; and (v) dense thick smooth. The AHC dendrogram is a multivariate cluster analysis examining the relationship between the biovolume, thickness and Ra measurements for the biofilms formed by each *S*. *epidermidis* isolate. The x-axis represents the dissimilarity score of merged clusters.

Among the 98 *S*. *epidermidis* isolates in Clade A, confocal microscopy (CLSM) imaging revealed that all isolates were adherent and formed biofilms *in vitro*, with microscale variability in their biofilm structure patterns (Fig 2A). Isolates adhered and formed patchy to multi-layered biofilms, with varying thicknesses from 3.42 μm (thin) to 30.97 μm (thick). Biofilm formation was consistent among three independent replicate assays (standard deviation values +/- 4 μm). Analysis of the Z-stacks using the Zen software orthogonal view and 3D imaging functions (Carl Zeiss, Jena, Germany) revealed that isolates either adhered and formed thick dense biofilms with an uneven topography (Fig 2Ai and 2Av) or patchy biofilms with different sized gaps between the bacterial aggregates (Fig 2Aii–2Aiv). COMSTAT software was then used to quantify the average biovolume (μm^3^ per μm^2^), average thickness (μm) and roughness coefficient (Ra; μm) of the CLSM Z-stacks from each isolate (S2 Table). These measurements support the visual observations of variation in biofilm morphotypes. T the largest biofilm biovolume and thickness measurements (>10 μm^3^ per μm^2^ and >10 μm^2^ respectively) were seen in isolates that formed thick dense biofilms with either a smooth or uneven topography (Fig 2Ai and 2Av). These werecompared to the thinner patchier biofilms (<10 μm^3^ per μm^2^ and <10 μm^2^ respectively; Fig 2Aii–2Aiv). Roughness coefficient (Ra) values were more varied falling into two groups, Ra <0.1 μm, smooth and homogenous, or Ra >0.1 μm, rough and heterogeneous. Regression analysis confirmed a correlation between biovolume and thickness but not with roughness coefficient (S1 Fig). AHC dendrogram is a multivariate cluster analysis using the biovolume, thickness and Ra measurements from each isolate, and clustered isolates together based on structure metrics (Fig 2B). Five biofilm morphotypes were identified based on the AHC dendogram. Isolates were classified as: dense thick uneven (29%), patchy thin smooth (21%), patchy thin uneven (18%), patchy thin rough (18%), and dense thick smooth (13%).

**Fig 3:**
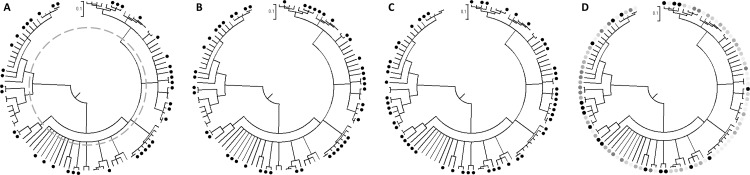
rMLST CLONALFRAME genealogies of clade A, showing COMSTAT results: A) Biovolume: black circle >10 μm3 per μm2; B) Thickness, black circle >10 μm; C) Roughness coefficient (Ra), black circle >0.10 μm; and D) Morphotypes, patchy thin rough (black circle), dense thick smooth (dark grey circle), patchy thin uneven (grey circle), patchy thin smooth (light grey circle), dense thick uneven (pale grey circle). The scale bar (0.1) indicates the genetic distance in coalescent units and represents the number of substitutions per site. Dashed line in image A at 0.3 coalescent units was used to characterise 19 lineages.

Biofilm morphotype, biovolume, thickness and roughness coefficient, were mapped onto the Clade A rMLST CLONALFRAME tree (Fig 3). All 19 multi-ST lineages contained isolates with one or more biofilm morphotype. Isolates producing biovolumes >10 μm^3^ per μm^2^ were present in 12/19 lineages, with the percentage of high biovolume morphotypes per cluster ranging from 22% to 100%. Isolates producing biofilm thickness >10 μm were present in 13/19 clusters and the proportion of thick biofilms ranged from 22% to 100%. Isolates producing rough biofilms (Ra>0.1) were present in 14/19 clusters and the proportion of rough morphotypes per cluster ranged from 40% to 100%.

For each morphotype metric, chi-square tests for homogeneity were carried out to test the null hypothesis that populations (lineages) are homogeneous in their biofilm phenotypes. In the case of biovolume, roughness coefficient or morphotype (*p* = 0.22, 0.72 and 0.23 respectively), there was no evidence to reject the null hypothesis. In the case of biofilm thickness (*p* = 0.05) there was a significant difference in the incidence of thick biofilms in certain lineages, with thick biofilms being more common among CC2 isolates, accounting for 20% of the 98 isolates.

### Variation in the presence of known biofilm-associated genes

**Fig 4:**
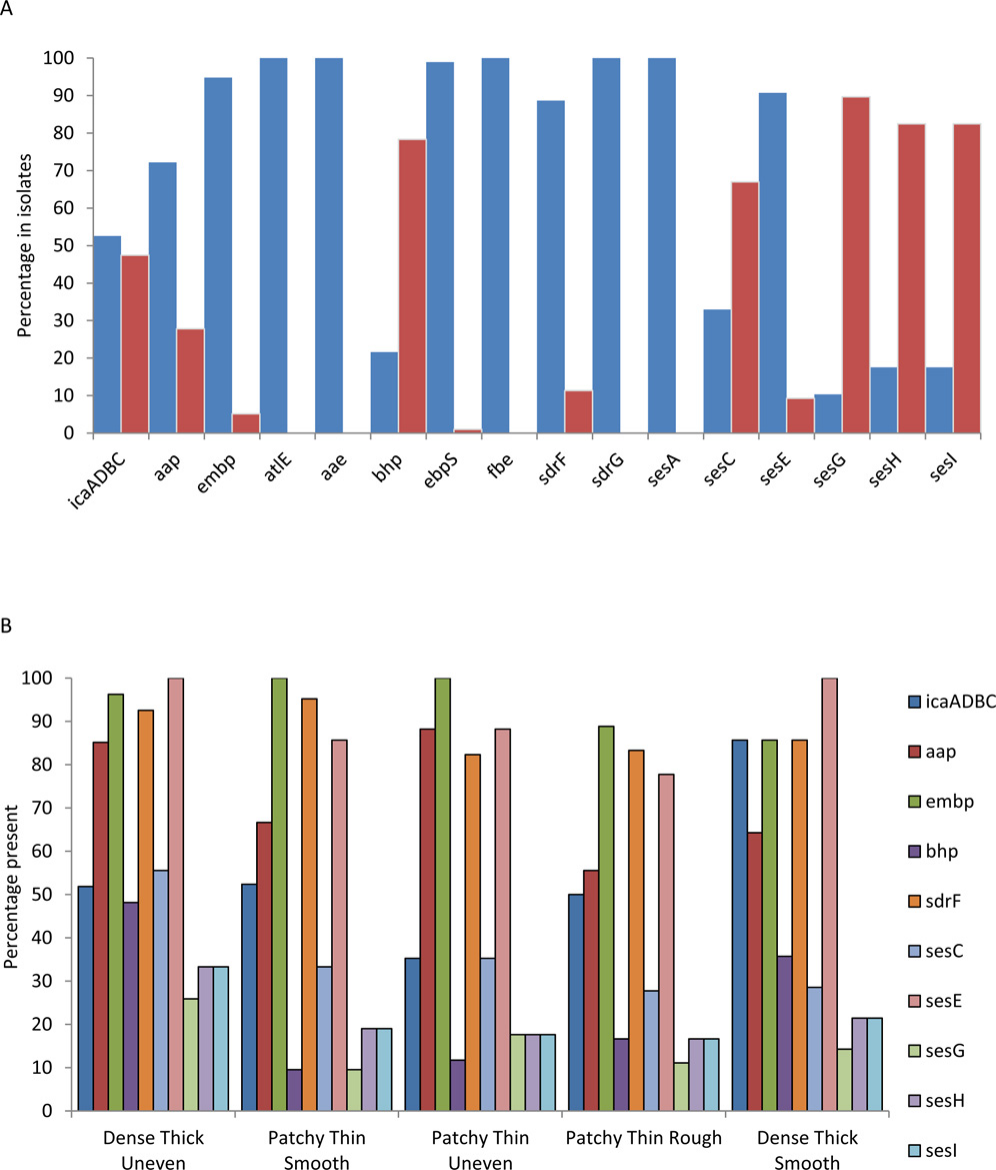
Graphs showing A) the presence and absence of the previously described adhesion and biofilm associated genes (9–16, 33) in clade A isolates, present (blue), absent (red); and B) percentage genes present in the isolates based on the 5 biofilm morphotypes.

The 98 genomes from clade A were analysed for the presence of genes previously described as being involved in adhesion [9–16, 33]. *bhp*, *sdrF*, *sesC*, *sesE*, *sesG*, *sesH* and *sesI* genes were present in 20%, 89%, 32%, 91%, 10%, 17% and 17% of the isolates respectively, whilst *atlE*, *aae*, *ebpS*, and *fbe*/*sdrG* were present in all the isolates (Fig 4A and S2 Table). The loci *icaADBC*, *aap* and *embp* known to be involved in biofilm formation [13–16] were present in 52%, 71% and 95% of the isolates respectively. The gene *mecA* was also identified in 56% of the isolates, with 82% of which were from a pathogenic source (results not shown). The presence of these genes was examined in relation to the 5 biofilm morphotypes (Fig 4B). The *icaADBC* cluster was most common in isolates that produced the dense thick rough morphotype (64%) and least common in those producing the patchy thick smooth (33%), whilst 93% of the dense thick rough morphotypes contained the *aap* gene compared to only 62% in the patchy thin rough morphotype. *embp* was found in 100% of the patchy thick smooth morphotypes versus 96% in the patchy thin rough, patchy thin smooth and dense thick smooth, and only in 86% of the dense thick rough morphotypes. The *bhp* and *sesE* genes were more associated with the patchy thick smooth biofilm morphotype (38% and 100%, respectively) than with the thinner biofilm morphotypes (19% and 81%, respectively); whilst the *sesG*, sesH, and *sesI* genes were present in higher numbers (14%, 43% and 43%, respectively) in the dense thick rough morphotype isolates compared to 8% and 4% respectively in the patchy thin rough morphotype isolates. Despite these differences there was no significant difference in the distribution of genes within the 5 biofilm patterns (*p* = 0.406).

**Fig 5:**
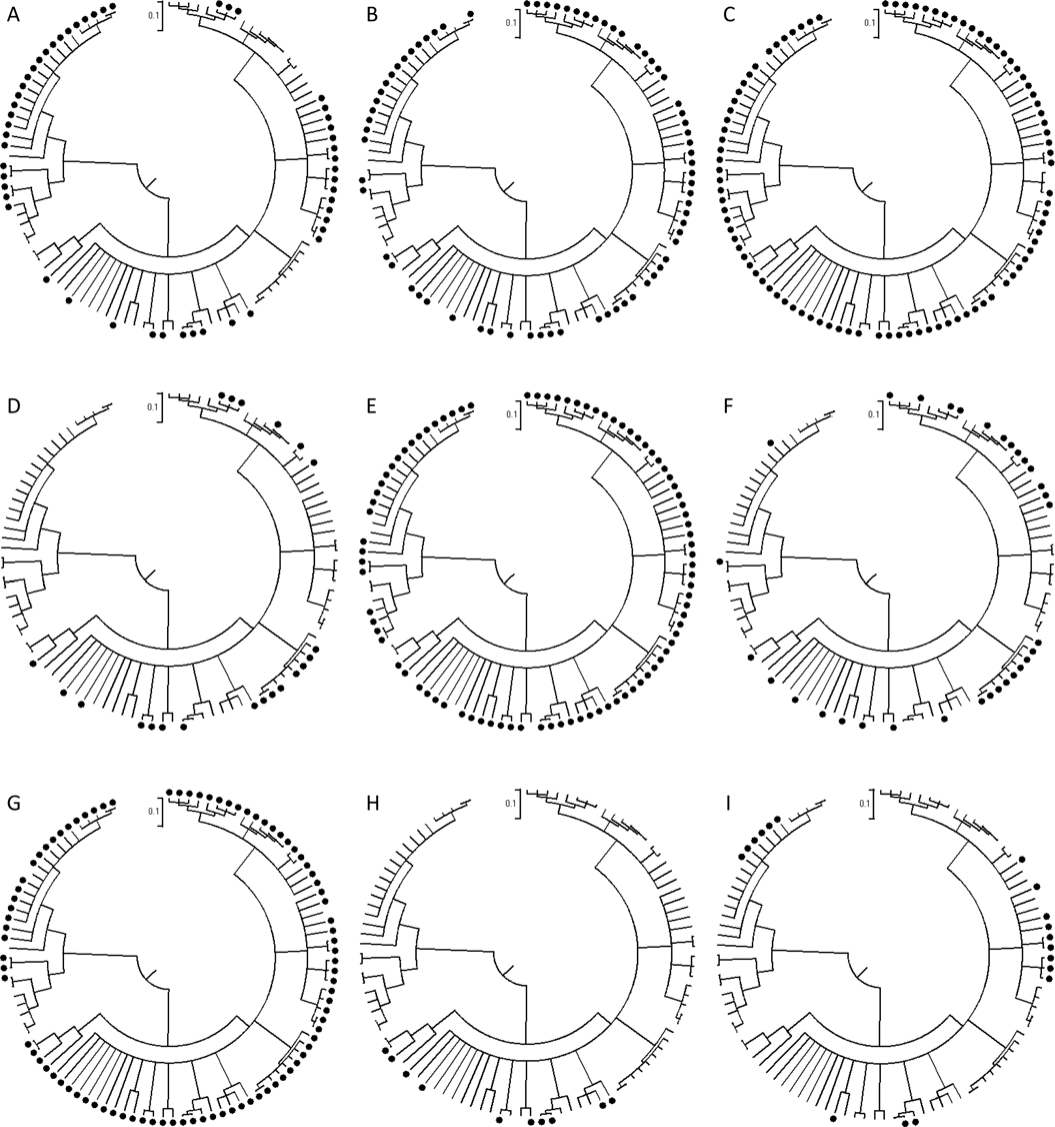
rMLST CLONALFRAME genealogies of Clade A showing the presence of specific genes in the isolates: (A) *icaADBC*, (B) *aap*, (C) *embp*, (D) *bhp*, (E) *sdrF*, (F) *sesC*, (G) s*esE*, (H) *sesG*, (I) *sesH/ sesI*. *atlE*, *aae*, *ebpS* and *fbe/sdrG* were present in all isolates. The scale bar (0.1) indicates the genetic distance in coalescent units.

The distribution of *icaADBC*, *aap*, *embp*, *bhp*, *sdrF*, *sesC*, *sesE*, *sesG*, *sesH* and *sesI* in clade A were mapped onto the rMLST CLONALFRAME tree (Fig 5). Distributions of the different genes on the tree varied depending on the gene, and correspond to the presence and absence data in Fig 4. Statistical analysis of the distribution of genes on the different sub-clades using Fisher’s Exact test and Chi-square, suggested a strong clade association for *icaADBC*, *aap*, *bhp*, *sdrF*, *sesC*, *sesE*, *sesG*, *sesH*, and *sesI* (*p* ≤ 0.05; S1 Table) but not for *embp* (*p* = 0.55). The allele numbers for each locus were analysed. With the exception of *aap* and *bhp* which showed sub-clade specific allelic variation, identical alleles were present in (2–14) sub-clades. This is consistent with the action of multiple recombination at these loci.

## Discussion

*S*. *epidermidis* infections associated with biomedical implants have become much more common with the increased use of biomaterials in medicine. In recent years, some studies have shown that commensal *S*. *epidermidis* populations differ from disease-causing strains in the frequency of the carriage of virulence factors such as those involved in adhesion and biofilm formation [18, 35–38]. The genealogical reconstruction of *S*. *epidermidis* genotypes from commensal and pathogenic sources showed evidence of clusters of related isolates consistent with clade and clonal complex designations previously described using whole genome sequencing and MLST [6, 32]. This provided the basis for testing if variation in adhesion and biofilm formation could mirror the genetic structure and distinguish virulent bacterial strains as particular lineages.

The combination of fine-scale phenotypic information with genotype information can provide insight into the evolution of *S*. *epidermidis*. In some bacteria for instance those that occupy multiple niches, genetic structuring of the population can reflect phenotypic differences. For example, in *Campylobacter jejuni*, different clonal complexes can dominate in different animal hosts [39], and in *Helicobacter pylori* population structure of the bacteria reflects the country of origin of the human from which it was isolated [40], whilst in *S*. *aureus* different clonal complexes are specifically associated with epidemic clones from specific countries [41, 42]. This niche segregation can be used to explain genetic structure in a population, according to an evolutionary model where isolates become separated in different niches and their genomes progressively diversify over time, accompanied by the accumulation of amino acid differences and concomitant phenotypic diversification.

Under this evolutionary model, *S*. *epidermidis* sequence clusters should display similar phenotypes. The biofilm phenotype is complex, incorporating live and dead bacteria [43]. Biofilms can be considered a two-step process involving primary attachment of bacterial cells to a surface via adhesins, followed by accumulation of the attached bacteria into a multi-layered biofilm via intercellular interactions [44]. When conditions are such that the ability to adhere and form a biofilm confers a strong selective advantage, for example on an implant, lineages that trace ancestry to strong biofilm formers will increase as a proportion of the population [45]. Under these circumstances, a phylogenetic tree will show clusters of biofilm forming lineages that have expanded clonally. However, while there was some evidence of sequence clusters of isolates that form thick biofilms (*p* = 0.05), statistical analyses of the CLONALFRAME tree showed random clustering of other biofilm variables and morphotypes in relation to clade distribution. There are a number of possible reasons for this. First, the confocal microscopy-derived biofilm variables contain less phenotypic information than is required for discrimination at this level. An explanation for this is that the biovolume, thickness and roughness variables mask other more informative biofilm-associated variables. Second, it may also be that the evolutionary model of linked genotypic and phenotypic diversification is too simplistic, particularly when applied to bacteria, such as *S*. *epidermidis*, that can undergo transition between multiple niches.

Some of the genes involved in biofilm formation have been well studied in *S*. *epidermidis*, but little is known about variation in gene presence among genetically diverse populations [33]. Biofilm-associated genes including *atlE*, *aae*, *ebpS*, and *fbe*/*sdrG* were found in all isolates in this study, and *icaADBC*, *aap*, *embp*, *bhp*, *sdrF*, *sesC*, *sesE*, *sesG*, *sesH* and *sesI* showed variation between the isolates and clades. The presence of *aap* or *sesE* showed a significant association with biofilm. The accumulation-associated protein (Aap) encoded by *aap* is known to be involved in biofilm formation, whilst an actual function for SesE, a cell wall surface anchor protein like Aap, has yet to be elucidated [10, 28]. However, the results in this study suggest the *sesE* gene product may have some involvement in biofilm accumulation.

The role of other biofilm associated genes in biofilm morphotype variation may be more complex. For example, PIA, encoded by the *ica* genes, is the most common molecule associated with biofilm formation in *S*. *epidermidis* [13, 14] and it has been suggested that the absence of the *ica operon* is more common in commensal *S*. *epidermidis* strains [46, 47]. However, the results presented here, show that the PIA-independent *aap* gene, that also mediates primary adhesion and/or biofilm accumulation [15, 48], was more frequently found in both pathogenic and commensal isolates than the *ica* operon, which is consistent with other studies [28, 49] and a complex interaction among biofilm associated genes that has been previously described and which may involve: (i) switching from PIA production to Aap mechanisms resulting in differences in biofilm substructures [50]; (ii) a role for Aap in anchoring PIA to the cell surface [51]; (iii) phase variation and the ‘switching on and off’ of adhesion and biofilm formation genes or other mechanisms of transcriptional regulation [52–54]. All of these factors make establishing a direct link between biofilm genes and phenotype variation more difficult.

The presence of genes associated with adhesion and biofilm formation in divergent rMLST sequence clusters (Fig 4) is evidence for horizontal gene transfer. Genome variation in *S*. *epidermidis* is generally thought to be generated more often by homologous recombination than by point mutation [55] compared to *S*. *aureus* [32, 56]. In addition, as in *S*. *aureus*, much of the genetic variation between *S*. *epidermidis* strains is associated with accessory genome variation in genomic islands, phage elements, and integrated plasmids [28, 46]. This can have major implications for resistance and virulence development in *S*. *epidermidis* [28]. Thus the mobility of adhesion and biofilm associated genes, including those in the *ica* operon [55], may be important in the increased abundance of phenotypes that aid adaptation to novel environments, and the transition from the commensal environment to infection.

## Conclusion

This study highlights the genetic diversity of *S*. *epidermidis* isolates from commensal and clinical samples and demonstrates the widespread capacity of these bacteria to adhere and form multilayer biofilms in *in vitro* assays. Biofilm phenotypes were highly variable and could be classified into various morphotypes. However, these did not correlate well with the clonal frame of the *S*. *epidermidis* isolates. Furthermore, while there was some correlation between genotypic groupings (clade, clonal complex and sequence type) and known biofilm-associated genes, there was also evidence for the widespread acquisition and mobility of these genes between lineages. Taken together, these findings are consistent with a multifactorial cell-cell adhesion processes resulting in different biofilm structures, involving complex adaptive mechanisms potentially involving multiple determinants of biofilm formation.

## Supporting Information

S1 Fig **Regression analysis graphs: A) biovolume versus thickness; B) biovolume versus roughness coefficient (Ra); and C) thickness versus roughness coefficient (Ra).** The results show a correlation between biovolume and thickness but not with roughness coefficient.
(TIF)

S1 Table Statistical analysis results using Fisher’s exact. Level of significance was set at *p* ≤ 0.05; statistically significant samples are in bold.
(DOCX)

S2 Table Summary of the Clade distribution, Clonal Complex results, COMSTAT results and biofilm structure pattern analysis from of the 98 isolates in Clade A (1 = present, 0 = absent). (DOCX)
