# Correction: Biofilm Morphotypes and Population Structure among *Staphylococcus epidermidis* from Commensal and Clinical Samples

**DOI:** 10.1371/journal.pone.0154510

**Published:** 2016-04-21

**Authors:** Llinos G. Harris, Susan Murray, Ben Pascoe, James Bray, Guillaume Meric, Leonardos Mageiros, Thomas S. Wilkinson, Rose Jeeves, Holger Rohde, Stefan Schwarz, Herminia de Lencastre, Maria Miragaia, Joana Rolo, Rory Bowden, Keith A. Jolley, Martin C. J. Maiden, Dietrich Mack, Samuel K. Sheppard

The sixth author’s name is spelled incorrectly. The correct name is: Leonardos Mageiros. The correct citation is: Llinos G. Harris, Susan Murray, Ben Pascoe, James Bray, Guillaume Meric, Leonardos Mageiros, Thomas S. Wilkinson, Rose Jeeves, Holger Rohde, Stefan Schwarz, Herminia de Lencastre, Maria Miragaia, Joana Rolo, Rory Bowden, Keith A. Jolley, Martin C. J. Maiden, Dietrich Mack, Samuel K. Sheppard (2016) Biofilm Morphotypes and Population Structure among *Staphylococcus epidermidis* from Commensal and Clinical Samples. PLoS ONE 11(3): e0151240. doi: 10.1371/journal.pone.0151240
