# Supplementary figures and images for: Biofilm Morphotypes and Population Structure among Staphylococcus epidermidis from Commensal and Clinical Samples
Source: PLoS One. 2016 Mar 15;11(3):e0151240. doi: 10.1371/journal.pone.0151240 (PMC4792440; doi:10.1371/journal.pone.0151240)

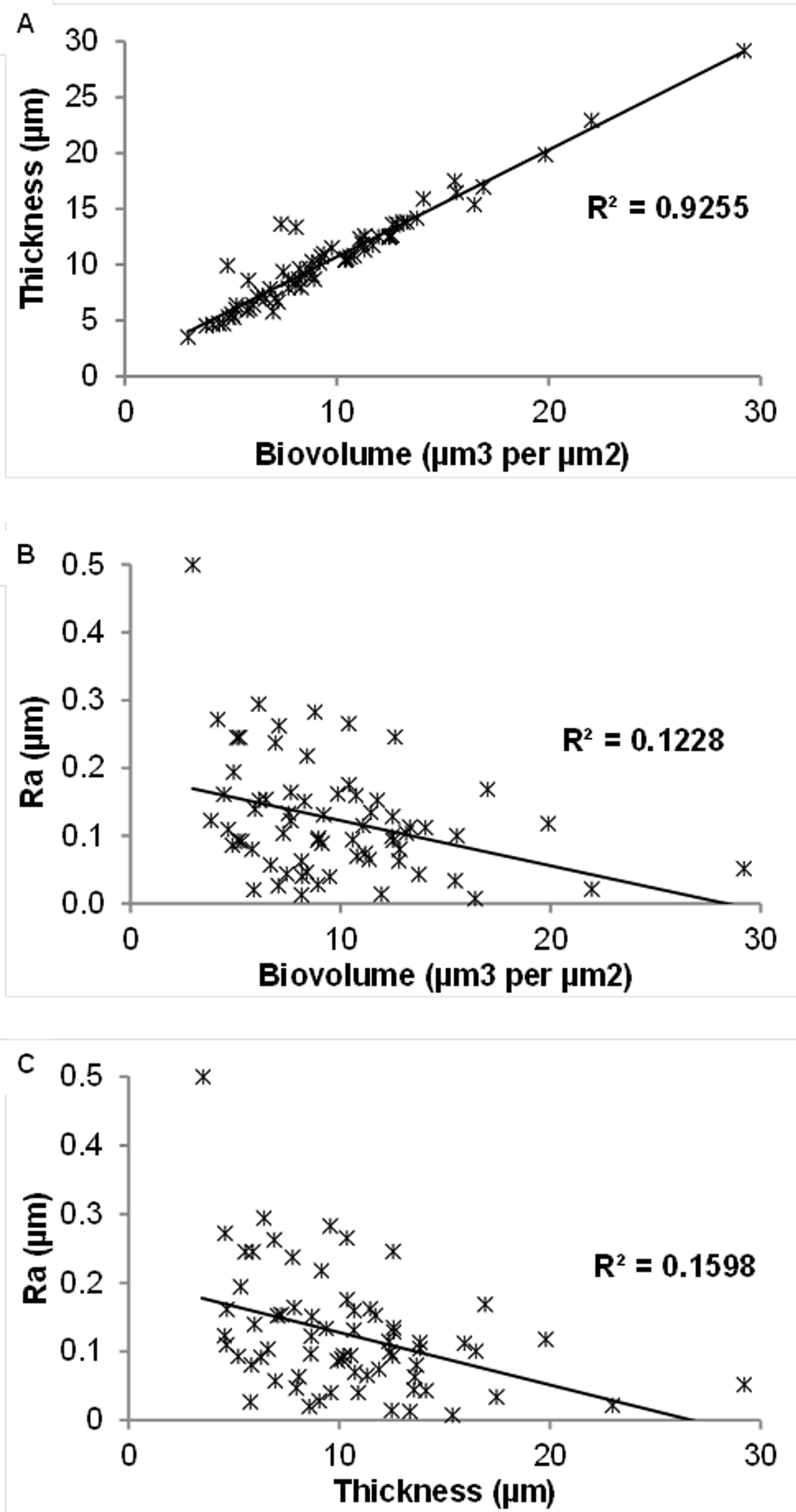

Supplement: S1 Fig — Regression analysis graphs: A) biovolume versus thickness; B) biovolume versus roughness coefficient (Ra); and C) thickness versus roughness coefficient (Ra). The results show a correlation between biovolume and thickness but not with roughness coefficient. (TIF) [file pone.0151240.s001.tif]
