# Supplementary material for: Biofilm Morphotypes and Population Structure among Staphylococcus epidermidis from Commensal and Clinical Samples
Source: PLoS One. 2016 Mar 15;11(3):e0151240. doi: 10.1371/journal.pone.0151240 (PMC4792440; doi:10.1371/journal.pone.0151240)
Supplement: S1 Table — Level of significance was set at p ≤ 0.05; statistically significant samples are in bold. (DOCX) [file pone.0151240.s002.docx]

S1 Table. Statistical analysis results using Fisher’s exact. Level of significance was set at p ≤ 0.05; statistically significant samples are in bold.

| Phenotype | Phenotype vs. clade A | | |  |  |  |  |  |  |  |  |
| --- | --- | --- | --- | --- | --- | --- | --- | --- | --- | --- | --- |
| Biovolume | 0.22 | | |  |  |  |  |  |  |  |  |
| Thickness | **0.05** | | |  |  |  |  |  |  |  |  |
| Ra | 0.72 | | |  |  |  |  |  |  |  |  |
|  |  |  | | | |  | |  | |  | |
| Genes | % present in isolates (n =98) | | Gene vs. clade A | | Gene vs. Biovolume | | Gene vs. Thickness | | Gene vs. Ra | |  |
| *icaADBC* | 49 | | **< 0.001** | | 0.3 | | 1 | | 0.54 | |  |
| *aap* | 71 | | **0.002** | | 1 | | **0.04** | | 0.37 | |  |
| *embp* | 95 | | 0.55 | | 1 | | 0.65 | | 1 | |  |
| *atlE* | 100 | | 1 | | 1 | | 1 | | 1 | |  |
| *aae* | 100 | | 1 | | 1 | | 1 | | 1 | |  |
| *bhp* | 20 | | **< 0.001** | | 0.21 | | 0.21 | | 1 | |  |
| *ebpS* | 99 | | 1 | | 1 | | 0.43 | | 0.44 | |  |
| *fbe* | 100 | | 1 | | 1 | | 1 | | 1 | |  |
| *sdrF* | 89 | | **< 0.001** | | 0.18 | | 0.59 | | 0.78 | |  |
| *sdrG* | 100 | | 1 | | 1 | | 1 | | 1 | |  |
| *sesA* | 100 | | 1 | | 1 | | 1 | | 1 | |  |
| *sesC* | 100 | | 1 | | 1 | | 1 | | 1 | |  |
| *sesE* | 91 | | **< 0.001** | | 0.147 | | **0.034** | | 0.162 | |  |
| *sesG* | 11 | | **< 0.001** | | 0.521 | | 0.534 | | 1 | |  |
| *sesH* | 100 | | 1 | | 1 | | 1 | | 1 | |  |
| *sesI* | 17 | | 0.07 | | 0.253 | | 0.43 | | 0.44 | |  |
| *mecA* | 56 | | **< 0.001** | | 0.41 | | 0.15 | | 0.84 | |  |
