# Supplementary material for: Biofilm Morphotypes and Population Structure among Staphylococcus epidermidis from Commensal and Clinical Samples
Source: PLoS One. 2016 Mar 15;11(3):e0151240. doi: 10.1371/journal.pone.0151240 (PMC4792440; doi:10.1371/journal.pone.0151240)
Supplement: S2 Table — (DOCX) [file pone.0151240.s003.docx]

**S2 Table.** Summary of the Clade distribution, Clonal Complex results, COMSTAT results and biofilm structure pattern analysis from of the 98 isolates in Clade A (1= present, 0 = absent).

| **Clade** | **Source** | **Epidemiology** | **Biovolume (µm3 per µm2 )** | **Thickness (µm)** | **Ra (µm)** | **Biofilm morphotype** | ***ica ADBC*** | ***aap*** | ***embp*** | ***atlE*** | ***aae*** | ***bhp*** | ***ebpS*** | ***fbe*** | ***sdrF*** | ***sdrG*** | ***sesC*** | ***sesE*** | ***sesG*** | ***sesH*** | ***sesI*** |
| --- | --- | --- | --- | --- | --- | --- | --- | --- | --- | --- | --- | --- | --- | --- | --- | --- | --- | --- | --- | --- | --- |
| 1 | nasal | commensal | 5.2 | 5.97 | 0.24 | patchy thin rough | 0 | 1 | 1 | 1 | 1 | 0 | 1 | 1 | 1 | 1 | 1 | 1 | 0 | 0 | 0 |
| 1 | nasal | commensal | 8.12 | 13.34 | 0.01 | patchy thick smooth | 0 | 1 | 1 | 1 | 1 | 0 | 1 | 1 | 1 | 1 | 0 | 1 | 0 | 0 | 0 |
| 1 | nasal | commensal | 8.73 | 9.64 | 0.28 | patchy thin rough | 0 | 1 | 1 | 1 | 1 | 0 | 1 | 1 | 1 | 1 | 0 | 1 | 0 | 0 | 0 |
| 1 | skin | commensal | 6.09 | 6.37 | 0.29 | patchy thin rough | 0 | 1 | 1 | 1 | 1 | 0 | 1 | 1 | 1 | 1 | 1 | 1 | 0 | 0 | 0 |
| 1 | PJI | pathogenic | 29.17 | 29.17 | 0.05 | dense thick smooth | 1 | 1 | 1 | 1 | 1 | 1 | 1 | 1 | 1 | 1 | 1 | 1 | 0 | 0 | 0 |
| 1 | PJI | pathogenic | 19.86 | 19.86 | 0.12 | dense thick smooth | 1 | 1 | 1 | 1 | 1 | 1 | 1 | 1 | 1 | 1 | 1 | 1 | 0 | 0 | 0 |
| 1 | nasal | commensal | 13.73 | 14.14 | 0.04 | dense thick smooth | 1 | 1 | 1 | 1 | 1 | 1 | 1 | 1 | 1 | 1 | 0 | 1 | 0 | 0 | 0 |
| 1 | skin | commensal | 4.83 | 9.9 | 0.09 | patchy thin smooth | 0 | 1 | 1 | 1 | 1 | 0 | 1 | 1 | 1 | 1 | 0 | 1 | 0 | 0 | 0 |
| 1 | PJI | pathogenic | 7.47 | 9.36 | 0.13 | patchy thin smooth | 0 | 1 | 1 | 1 | 1 | 0 | 1 | 1 | 1 | 1 | 1 | 1 | 0 | 0 | 0 |
| 1 | PJI | pathogenic | 5.33 | 6.36 | 0.09 | patchy thin smooth | 0 | 1 | 1 | 1 | 1 | 0 | 1 | 1 | 1 | 1 | 1 | 1 | 0 | 0 | 0 |
| 1 | animal | commensal | 11.98 | 12.5 | 0.01 | dense thick smooth | 0 | 0 | 1 | 1 | 1 | 1 | 1 | 1 | 1 | 1 | 0 | 1 | 0 | 0 | 0 |
| 1 | skin | commensal | 6.61 | 6.93 | 0.06 | patchy thin smooth | 0 | 1 | 1 | 1 | 1 | 0 | 1 | 1 | 1 | 1 | 1 | 1 | 0 | 0 | 0 |
| 1 | skin | commensal | 9.84 | 11.48 | 0.16 | patchy thick rough | 0 | 1 | 1 | 1 | 1 | 0 | 1 | 1 | 1 | 1 | 0 | 1 | 0 | 0 | 0 |
| 1 | nasal | commensal | 7.42 | 13.62 | 0.04 | patchy thin rough | 0 | 1 | 1 | 1 | 1 | 0 | 1 | 1 | 1 | 1 | 0 | 1 | 0 | 0 | 0 |
| 1 | nasal | commensal | 8.34 | 7.95 | 0.05 | patchy thin smooth | 0 | 1 | 1 | 1 | 1 | 1 | 1 | 1 | 1 | 1 | 1 | 1 | 0 | 0 | 0 |
| 1 | animal | commensal | 9.44 | 10.96 | 0.04 | patchy thick smooth | 0 | 1 | 1 | 1 | 1 | 0 | 1 | 1 | 1 | 1 | 1 | 1 | 0 | 0 | 0 |
| 1 | PJI | pathogenic | 10.68 | 10.68 | 0.16 | dense thick rough | 0 | 1 | 1 | 1 | 1 | 1 | 1 | 1 | 1 | 1 | 1 | 1 | 0 | 1 | 1 |
| 1 | skin | commensal | 4.46 | 4.71 | 0.16 | patchy thin rough | 0 | 0 | 1 | 1 | 1 | 0 | 1 | 1 | 1 | 1 | 0 | 1 | 0 | 0 | 0 |
| 1 | skin | commensal | 3.81 | 4.5 | 0.12 | patchy thin smooth | 0 | 0 | 1 | 1 | 1 | 0 | 1 | 1 | 1 | 1 | 1 | 1 | 0 | 0 | 0 |
| 2 | catheter | pathogenic | 14.04 | 15.9 | 0.11 | dense thick rough | 1 | 1 | 1 | 1 | 1 | 0 | 1 | 1 | 1 | 1 | 0 | 1 | 0 | 0 | 0 |
| 2 | catheter | pathogenic | 5.77 | 5.89 | 0.08 | patchy thin smooth | 1 | 1 | 1 | 1 | 1 | 0 | 1 | 1 | 1 | 1 | 0 | 1 | 0 | 0 | 0 |
| 2 | catheter | pathogenic | 7.69 | 7.9 | 0.16 | patchy thin rough | 1 | 1 | 1 | 1 | 1 | 0 | 1 | 1 | 1 | 1 | 0 | 1 | 0 | 0 | 0 |
| 2 | catheter | pathogenic | 15.6 | 16.45 | 0.1 | dense thick rough | 1 | 1 | 1 | 1 | 1 | 0 | 1 | 1 | 1 | 1 | 0 | 1 | 0 | 0 | 0 |
| 2 | catheter | pathogenic | 13.29 | 13.81 | 0.11 | dense thick rough | 1 | 1 | 1 | 1 | 1 | 0 | 1 | 1 | 1 | 1 | 0 | 1 | 0 | 0 | 0 |
| 2 | PJI | pathogenic | 12.59 | 12.59 | 0.25 | dense thick rough | 1 | 1 | 0 | 1 | 1 | 0 | 1 | 1 | 1 | 1 | 0 | 1 | 0 | 1 | 1 |
| 2 | PJI | pathogenic | 4.72 | 4.72 | 0.11 | patchy thin smooth | 1 | 1 | 0 | 1 | 1 | 0 | 1 | 1 | 1 | 1 | 0 | 1 | 0 | 1 | 1 |
| 2 | catheter | pathogenic | 4.93 | 5.29 | 0.19 | patchy thin smooth | 1 | 1 | 1 | 1 | 1 | 0 | 1 | 1 | 1 | 1 | 0 | 1 | 0 | 1 | 1 |
| 2 | catheter | pathogenic | 13.15 | 13.79 | 0.1 | dense thick rough | 1 | 1 | 1 | 1 | 1 | 0 | 1 | 1 | 1 | 1 | 0 | 1 | 0 | 1 | 1 |
| 2 | catheter | pathogenic | 11.19 | 11.84 | 0.07 | dense thick smooth | 1 | 1 | 1 | 1 | 1 | 0 | 1 | 1 | 1 | 1 | 0 | 1 | 0 | 1 | 1 |
| 2 | other | pathogenic | 11.39 | 12.56 | 0.13 | patchy thin rough | 0 | 0 | 1 | 1 | 1 | 0 | 1 | 1 | 1 | 1 | 0 | 1 | 0 | 0 | 0 |
| 2 | PJI | pathogenic | 16.96 | 16.96 | 0.17 | dense thick rough | 1 | 1 | 1 | 1 | 1 | 0 | 1 | 1 | 1 | 1 | 0 | 1 | 0 | 1 | 1 |
| 2 | catheter | pathogenic | 8.12 | 8.16 | 0.06 | patchy thin smooth | 1 | 1 | 1 | 1 | 1 | 0 | 1 | 1 | 1 | 1 | 0 | 0 | 0 | 0 | 0 |
| 2 | skin | commensal | 12.84 | 13.65 | 0.08 | dense thick smooth | 1 | 1 | 1 | 1 | 1 | 0 | 1 | 1 | 1 | 1 | 1 | 1 | 0 | 0 | 0 |
| 2 | skin | commensal | 5.85 | 8.59 | 0.02 | patchy thin smooth | 1 | 1 | 1 | 1 | 1 | 0 | 1 | 1 | 1 | 1 | 1 | 1 | 0 | 1 | 1 |
| 3 | catheter | pathogenic | 11.13 | 12.3 | 0.11 | dense thick rough | 0 | 1 | 1 | 1 | 1 | 1 | 1 | 1 | 1 | 1 | 1 | 1 | 0 | 0 | 0 |
| 3 | catheter | pathogenic | 6.85 | 7.83 | 0.24 | patchy thin rough | 0 | 1 | 1 | 1 | 1 | 1 | 1 | 1 | 1 | 1 | 1 | 1 | 0 | 0 | 0 |
| 3 | PJI | pathogenic | 10.8 | 10.8 | 0.07 | dense thick smooth | 0 | 1 | 1 | 1 | 1 | 1 | 1 | 1 | 1 | 1 | 1 | 1 | 0 | 0 | 0 |
| 3 | PJI | pathogenic | 12.53 | 12.53 | 0.09 | dense thick smooth | 0 | 0 | 1 | 1 | 1 | 0 | 1 | 1 | 1 | 1 | 1 | 1 | 0 | 0 | 0 |
| 3 | PJI | pathogenic | 12.42 | 12.42 | 0.1 | dense thick smooth | 0 | 1 | 1 | 1 | 1 | 1 | 1 | 1 | 1 | 1 | 1 | 1 | 0 | 0 | 0 |
| 3 | PJI | pathogenic | 10.53 | 10.53 | 0.09 | patchy thick smooth | 0 | 1 | 1 | 1 | 1 | 1 | 1 | 1 | 1 | 1 | 1 | 1 | 0 | 0 | 0 |
| 3 | nasal | commensal | 8.9 | 10.25 | 0.09 | patchy thick smooth | 0 | 1 | 1 | 1 | 1 | 1 | 1 | 1 | 1 | 1 | 1 | 1 | 0 | 0 | 0 |
| 3 | nasal | commensal | 6.21 | 7.13 | 0.15 | patchy thin rough | 0 | 1 | 1 | 1 | 1 | 1 | 1 | 1 | 1 | 1 | 1 | 1 | 0 | 0 | 0 |
| 3 | other | pathogenic | 7.26 | 6.66 | 0.1 | patchy thick smooth | 0 | 0 | 1 | 1 | 1 | 0 | 1 | 1 | 1 | 1 | 1 | 1 | 0 | 0 | 0 |
| 4 | PJI | pathogenic | 7.08 | 6.98 | 0.26 | patchy thin smooth | 0 | 0 | 1 | 1 | 1 | 0 | 1 | 1 | 1 | 1 | 1 | 1 | 0 | 0 | 0 |
| 4 | skin | commensal | 8.29 | 8.71 | 0.15 | patchy thin rough | 1 | 1 | 1 | 1 | 1 | 0 | 1 | 1 | 1 | 1 | 0 | 1 | 1 | 0 | 0 |
| 4 | skin | commensal | 8.96 | 8.68 | 0.1 | patchy thin rough | 1 | 1 | 1 | 1 | 1 | 1 | 1 | 1 | 1 | 1 | 0 | 1 | 1 | 0 | 0 |
| 4 | nasal | commensal | 8.91 | 9.05 | 0.03 | patchy thin smooth | 1 | 1 | 1 | 1 | 1 | 0 | 1 | 1 | 1 | 1 | 0 | 1 | 1 | 1 | 1 |
| 5 | animal | commensal | 7.02 | 5.77 | 0.03 | patchy thin smooth | 1 | 1 | 1 | 1 | 1 | 1 | 1 | 1 | 1 | 1 | 0 | 1 | 1 | 0 | 0 |
| 5 | animal | commensal | 9.25 | 10.7 | 0.13 | patchy thin smooth | 1 | 1 | 1 | 1 | 1 | 0 | 1 | 1 | 1 | 1 | 0 | 1 | 0 | 1 | 1 |
| 5 | PJI | pathogenic | 11.71 | 11.71 | 0.15 | patchy thick smooth | 0 | 0 | 1 | 1 | 1 | 0 | 1 | 1 | 1 | 1 | 0 | 1 | 0 | 0 | 0 |
| 5 | nasal | commensal | 5.08 | 5.54 | 0.24 | dense thick rough | 1 | 1 | 1 | 1 | 1 | 0 | 1 | 1 | 0 | 1 | 1 | 1 | 1 | 0 | 0 |
| 6 | skin | commensal | 8.48 | 9.11 | 0.22 | patchy thin rough | 0 | 0 | 1 | 1 | 1 | 1 | 1 | 1 | 1 | 1 | 0 | 1 | 0 | 0 | 0 |
| 6 | PJI | pathogenic | 12.55 | 12.55 | 0.13 | patchy thin rough | 0 | 1 | 0 | 1 | 1 | 1 | 1 | 1 | 1 | 1 | 0 | 1 | 0 | 0 | 0 |
| 7 | catheter | pathogenic | 22.02 | 22.93 | 0.02 | dense thick rough | 0 | 0 | 1 | 1 | 1 | 1 | 1 | 1 | 1 | 1 | 1 | 1 | 1 | 0 | 0 |
| 7 | PJI | pathogenic | 10.41 | 10.41 | 0.26 | dense thick smooth | 0 | 1 | 1 | 1 | 1 | 0 | 1 | 1 | 1 | 1 | 0 | 1 | 0 | 0 | 0 |
| 8 | skin | commensal | 5.17 | 5.26 | 0.09 | patchy thin rough | 0 | 1 | 1 | 1 | 1 | 0 | 1 | 1 | 1 | 1 | 1 | 1 | 0 | 1 | 1 |
| 8 | catheter | pathogenic | 6.44 | 7.26 | 0.15 | patchy thin smooth | 1 | 0 | 1 | 1 | 1 | 0 | 1 | 1 | 1 | 1 | 0 | 1 | 0 | 0 | 0 |
| 9 | nasal | commensal | 12.71 | 13.58 | 0.06 | patchy thin smooth | 0 | 0 | 1 | 1 | 1 | 0 | 1 | 1 | 1 | 1 | 1 | 1 | 0 | 0 | 0 |
| 10 | nasal | commensal | 8.24 | 9.61 | 0.04 | patchy thin rough | 0 | 1 | 1 | 1 | 1 | 0 | 1 | 1 | 0 | 1 | 0 | 1 | 0 | 0 | 0 |
| 11 | animal | commensal | 15.52 | 17.5 | 0.03 | patchy thin smooth | 0 | 0 | 1 | 1 | 1 | 1 | 1 | 1 | 1 | 1 | 0 | 1 | 0 | 0 | 0 |
| 12 | animal | commensal | 9.18 | 10.17 | 0.09 | dense thick smooth | 0 | 0 | 1 | 1 | 1 | 0 | 1 | 1 | 1 | 1 | 1 | 1 | 0 | 0 | 0 |
| 13 | animal | commensal | 16.46 | 15.38 | 0.01 | patchy thick smooth | 1 | 1 | 1 | 1 | 1 | 1 | 1 | 1 | 1 | 1 | 0 | 1 | 1 | 0 | 0 |
| 14 | nasal | commensal | 7.68 | 8.65 | 0.12 | dense thick smooth | 0 | 1 | 1 | 1 | 1 | 0 | 1 | 1 | 1 | 1 | 0 | 1 | 0 | 0 | 0 |
| 15 | skin | commensal | 5.95 | 6.05 | 0.14 | dense thick smooth | 0 | 1 | 1 | 1 | 1 | 0 | 1 | 1 | 0 | 1 | 1 | 1 | 1 | 0 | 0 |
| 16 | PJI | pathogenic | 10.39 | 10.39 | 0.18 | patchy thin smooth | 0 | 1 | 1 | 1 | 1 | 0 | 1 | 1 | 0 | 1 | 1 | 1 | 1 | 0 | 0 |
| 17 | PJI | pathogenic | 11.32 | 11.32 | 0.07 | dense thick rough | 0 | 1 | 1 | 1 | 1 | 0 | 1 | 1 | 0 | 1 | 0 | 1 | 0 | 0 | 0 |
| 17 | skin | commensal | 4.11 | 4.63 | 0.27 | dense thick smooth | 0 | 0 | 1 | 1 | 1 | 1 | 1 | 1 | 1 | 1 | 0 | 1 | 1 | 0 | 0 |
| 17 | skin | commensal | 2.96 | 3.48 | 0.5 | patchy thin rough | 1 | 0 | 1 | 1 | 1 | 0 | 1 | 1 | 1 | 1 | 0 | 1 | 0 | 0 | 0 |
| 17 | PJI | pathogenic | 24.82 | 27.75 | 0.02 | patchy thin rough | 1 | 0 | 1 | 1 | 1 | 0 | 1 | 1 | 1 | 1 | 0 | 1 | 0 | 0 | 0 |
| 18 | PJI | pathogenic | 10.18 | 12.05 | 0.05 | dense thick smooth | 1 | 1 | 0 | 1 | 1 | 0 | 1 | 1 | 1 | 1 | 0 | 1 | 0 | 0 | 0 |
| 18 | catheter | pathogenic | 8.21 | 8.75 | 0.05 | patchy thick smooth | 1 | 0 | 1 | 1 | 1 | 0 | 1 | 1 | 1 | 1 | 0 | 1 | 0 | 0 | 0 |
| 18 | catheter | pathogenic | 8.47 | 10.17 | 0.2 | patchy thin smooth | 1 | 1 | 1 | 1 | 1 | 0 | 1 | 1 | 1 | 1 | 0 | 1 | 0 | 0 | 0 |
| 18 | catheter | pathogenic | 12.58 | 13.25 | 0.1 | patchy thin rough | 1 | 1 | 1 | 1 | 1 | 0 | 1 | 1 | 1 | 1 | 0 | 1 | 0 | 0 | 0 |
| 18 | nasal | commensal | 8.89 | 7.94 | 0.3 | dense thick rough | 1 | 1 | 1 | 1 | 1 | 0 | 1 | 1 | 1 | 1 | 0 | 1 | 0 | 1 | 1 |
| 18 | PJI | pathogenic | 24.87 | 24.87 | 0.07 | patchy thick smooth | 1 | 1 | 1 | 1 | 1 | 0 | 1 | 1 | 1 | 1 | 0 | 1 | 0 | 1 | 1 |
| 18 | PJI | pathogenic | 12.55 | 12.55 | 0.13 | dense thick smooth | 1 | 1 | 1 | 1 | 1 | 0 | 1 | 1 | 1 | 1 | 1 | 1 | 0 | 1 | 1 |
| 18 | catheter | pathogenic | 20.67 | 22.1 | 0.01 | dense thick rough | 1 | 1 | 0 | 1 | 1 | 0 | 1 | 1 | 1 | 1 | 0 | 1 | 0 | 1 | 1 |
| 18 | catheter | pathogenic | 9.32 | 9.27 | 0.06 | dense thick smooth | 1 | 1 | 1 | 1 | 1 | 0 | 1 | 1 | 1 | 1 | 0 | 1 | 0 | 1 | 1 |
| 18 | catheter | pathogenic | 14.25 | 14.87 | 0.1 | patchy thin smooth | 1 | 1 | 1 | 1 | 1 | 0 | 1 | 1 | 1 | 1 | 0 | 1 | 0 | 1 | 1 |
| 18 | catheter | pathogenic | 5.56 | 6.19 | 0.33 | dense thick smooth | 1 | 1 | 1 | 1 | 1 | 0 | 1 | 1 | 1 | 1 | 0 | 0 | 0 | 0 | 0 |
| 18 | catheter | pathogenic | 10.1 | 10.52 | 0.15 | patchy thin rough | 1 | 1 | 1 | 1 | 1 | 0 | 1 | 1 | 1 | 1 | 0 | 1 | 0 | 0 | 0 |
| 18 | catheter | pathogenic | 7.57 | 7.82 | 0.08 | dense thick rough | 1 | 1 | 1 | 1 | 1 | 0 | 1 | 1 | 1 | 1 | 0 | 1 | 0 | 0 | 0 |
| 18 | catheter | pathogenic | 6.78 | 6.81 | 0.08 | patchy thin smooth | 1 | 1 | 1 | 1 | 1 | 0 | 1 | 1 | 1 | 1 | 0 | 1 | 0 | 0 | 0 |
| 18 | catheter | pathogenic | 7.37 | 8.25 | 0.14 | patchy thin smooth | 1 | 1 | 1 | 1 | 1 | 0 | 1 | 1 | 1 | 1 | 0 | 1 | 0 | 0 | 0 |
| 18 | catheter | pathogenic | 7.21 | 7.39 | 0.15 | patchy thin smooth | 1 | 1 | 1 | 1 | 1 | 0 | 1 | 1 | 0 | 1 | 0 | 1 | 0 | 0 | 0 |
| 18 | nasal | commensal | 18.33 | 20.34 | 0.1 | patchy thin smooth | 1 | 1 | 1 | 1 | 1 | 0 | 1 | 1 | 0 | 1 | 0 | 1 | 0 | 0 | 0 |
| 18 | skin | commensal | 26.24 | 30.97 | 0.05 | dense thick smooth | 1 | 0 | 1 | 1 | 1 | 0 | 1 | 1 | 1 | 1 | 0 | 1 | 0 | 0 | 0 |
| 18 | other | pathogenic | 8.31 | 8.99 | 0.14 | dense thick smooth | 0 | 0 | 1 | 1 | 1 | 0 | 1 | 1 | 1 | 1 | 0 | 0 | 0 | 0 | 0 |
| 18 | PJI | pathogenic | 6.76 | 6.76 | 0.2 | patchy thin rough | 0 | 0 | 1 | 1 | 1 | 0 | 1 | 1 | 1 | 1 | 0 | 0 | 0 | 0 | 0 |
| 19 | PJI | pathogenic | 4.42 | 4.42 | 0.19 | patchy thin rough | 0 | 0 | 1 | 1 | 1 | 0 | 1 | 1 | 1 | 1 | 0 | 0 | 0 | 0 | 0 |
| 19 | PJI | pathogenic | 7.27 | 7.27 | 0.06 | patchy thin rough | 0 | 0 | 1 | 1 | 1 | 0 | 1 | 1 | 1 | 1 | 0 | 0 | 0 | 0 | 0 |
| 19 | PJI | pathogenic | 4.79 | 4.79 | 0.27 | patchy thin smooth | 0 | 0 | 1 | 1 | 1 | 0 | 1 | 1 | 1 | 1 | 0 | 0 | 0 | 0 | 0 |
| 19 | PJI | pathogenic | 4.51 | 4.51 | 0.28 | patchy thin rough | 1 | 0 | 1 | 1 | 1 | 0 | 1 | 1 | 0 | 1 | 0 | 0 | 0 | 0 | 0 |
| 19 | PJI | pathogenic | 8.44 | 9.52 | 0.23 | patchy thin rough | 1 | 0 | 1 | 1 | 1 | 0 | 1 | 1 | 0 | 1 | 0 | 0 | 0 | 0 | 0 |
| 19 | PJI | pathogenic | 5.12 | 5.12 | 0.17 | patchy thin rough | 1 | 1 | 1 | 1 | 1 | 0 | 1 | 1 | 0 | 1 | 0 | 1 | 0 | 0 | 0 |
| 19 | PJI | pathogenic | 20.91 | 20.91 | 0.03 | patchy thin rough | 1 | 1 | 1 | 1 | 1 | 0 | 1 | 1 | 0 | 1 | 0 | 1 | 0 | 0 | 0 |
| 19 | skin | commensal | 19.87 | 22.27 | 0.05 | dense thick smooth | 1 | 0 | 1 | 1 | 1 | 0 | 1 | 1 | 1 | 1 | 1 | 1 | 0 | 0 | 0 |
| 19 | other | pathogenic | 5.38 | 6.68 | 0.26 | dense thick smooth | 1 | 0 | 1 | 1 | 1 | 0 | 1 | 1 | 1 | 1 | 0 | 1 | 0 | 0 | 0 |
